# Efficacy and Safety of Chinese Medicine Lianhua Qingwen for Treating COVID-19: An Updated meta-Analysis

**DOI:** 10.3389/fphar.2022.888820

**Published:** 2022-06-03

**Authors:** Yapeng Li, Peng Xiao, Nanyang Liu, Zhijie Zhang

**Affiliations:** ^1^ Rehabilitation Therapy Center, Luoyang Orthopedic Hospital of Henan Province, Orthopedic Hospital of Henan Province, Luoyang, China; ^2^ Medical Experimental Research Center, Orthopedic Institute of Henan Province, Luoyang, China; ^3^ Department of Geratology, Xiyuan Hospital, China Academy of Chinese Medical Sciences, Beijing, China

**Keywords:** COVID-19, Lianhua Qingwen, traditional Chinese medicine, systematic review, meta-analysis

## Abstract

The traditional Chinese medicine formula Lianhua Qingwen (LQ) combined with western medicine therapy is beneficial to coronavirus disease-19 (COVID-19), but there is still a lack of strong evidence-based. We conducted a systematic review and meta-analysis to evaluate the efficacy and safety of LQ combined with western medicine for patients with COVID-19. Seven databases (Chinese and English) were searched by two independent reviewers. Search for relevant keywords such as “Chinese medicine,” “Chinese herbal medicine,” and “Lianhua Qingwen” in the titles and abstracts of articles retrieved in the databases. Randomized controlled trials or case-control studies that reported sufficient data of participants before and after the intervention were included. Two researchers independently reviewed the studies and extracted the data. Fixed-or random-effect model was used to calculate the overall pooled risk estimates. Forest plots were generated to show pooled results. Seven studies involving 916 participants were included in the meta-analysis. Overall, compared with the control group, the total efficacy (OR = 2.23, 95% CI 1.56, 3.18), adverse events (OR = 0.42, 95% CI 0.18, 0.97), chest computed tomography manifestations (OR = 1.74, 95% CI 1.12, 2.72), and aggravation rate of conversion to severe cases (OR = 0.47, 95% CI 0.30, 0.75) of the intervention group were better. Moreover, the intervention group has an advantage over the control group in improving clinical symptoms (fever, cough, fatigue, chest tightness, shortness of breath, and expectoration) and shortening the fever duration (*p* < 0.05). Our findings indicate that LQ combined with western medicine may be more effective in treating COVID-19. However, due to the urgency of SARS-CoV-2 outbreaks leading to low methodological quality and not rigorous designs. This meta-analysis cannot draw clear conclusions. PROSPERO registration number: CRD42020190757

## 1 Introduction

Recently, the World Health Organization (WHO) identified a new coronavirus, which was first discovered in Wuhan, China at the end of December 2019, and named it the novel coronavirus disease (COVID-19) (Zhou F. et al., 2020). The severe acute respiratory syndrome coronavirus 2 (SARS-CoV-2) belongs to the β-CoV family and has a highly homologous sequence with SARS-CoV. Host cell infection is mediated by the binding of spike glycoprotein to angiotensin-converting enzyme 2 (ACE2) (Xiao et al., 2020). The COVID-19 is a sudden acute respiratory infectious disease that is susceptible regardless of gender and age, with high infectivity and high fatality rate, causing serious harm to public health (Wölfel et al., 2020). At least more than 200 countries in the world have discovered COVID-19 cases, forming a world pandemic (Chen et al., 2020). To data more than 370 million cases of COVID-19 have been reported worldwide and more than 5.6 million deaths (Organization., 2022). COVID-19 infection is characterized by flu-like symptoms, including fever, dry cough, and fatigue (Zhou P. et al., 2020; Lu et al., 2020; Wrapp et al., 2020). At present, there are no approved or validated drugs against the virus.

Traditional Chinese medicine (TCM) is an ancient treatment strategy through special diagnostic methods and a mixture of Chinese botanical drugs prescribed by Chinese herbalists depending on the patient’s syndrome to cure disease. The COVID-19 belongs to the plague in TCM with the etiology of epidemic factor exposure (Hu et al., 2021). Studies demonstrated that several candidates with possible antiviral effects and improve clinical symptoms in patients with COVID-19 have been explored (Luo et al., 2020; Qing et al., 2020). The effect may be related to blocking the proliferation and replication of virus particles (Ding et al., 2017). According to reports, Chinese herbal medicine has successfully treated the severe acute respiratory syndrome (SARS) in 2003, providing valuable experience for the COVID-19 epidemic (Hu et al., 2021). At the beginning of the virus epidemic, the Chinese government formulated a syndrome differentiation treatment plan for TCM against COVID-19 achieved remarkable success.

Existing evidence shows that compared with western medicine treatment, integrating Chinese and western medicine may have a better effect on COVID-19. Lianhua Qingwen (LQ), a manufactured product composed of more than a dozen Chinese herbal medicines, has been shown to significantly inhibit SARS-CoV-2 replication, change virus morphology, and confer anti-inflammatory activity *in vitro* (Runfeng et al., 2020). It is reported that LQ paired with western medicine could significantly ameliorate the clinical symptoms, promote lung inflammation absorption, and shorten the course of COVID-19 (Lv et al., 2020; Wang et al., 2020). However, the evidence is based on small sample and it is necessary to integrate to provide more convincing date. The present study aimed to integrate the existing evidence to evaluate the efficacy and safety of LQ paired with western medicine in the treatment of patients with COVID-19 so that we could inform clinical practice.

## 2 Materials and Methods

### 2.1 Search Strategy

The protocol of this study has been registered in the PROSPERO (registration number: CRD42020190757). The systematic review and meta-analysis were conducted according to the Preferred Reporting Items for Systematic Reviews and Meta-Analysis (PRISRM) extension for meta-analysis (Moher et al., 2009). Two independent reviewers searched the databases of the Chinese Biomedical Literature Database, China National Knowledge Infrastructure, Chinese Science and Technology Periodical Database, and Wanfang database, PubMed, Embase, and Cochrane Library from the date of conception to 1 June 2021. The terms used in the search were: “Chinese medicine”, “Chinese herbal medicine”, “Lianhua Qingwen”, “novel coronavirus infected pneumonia”, “COVID-19”, “SARS-CoV-2”, “Corona Virus Disease 2019”, “NCP”, “2019-nCOV”. The search words in the Chinese databases are translations of the above words. References from the latest reviews were also searched in case of missing potential eligible clinical trials. We have no restrictions on the search.

### 2.2 Selection Criteria

Inclusion studies set the following criteria: 1) Patients: ordinary patients who meet the diagnostic criteria for COVID-19; 2) Intervention: patients in the treatment group received LQ (eg. decoction, capsule, or granule) and combined with routine western treatments (eg. antibiotic, antiviral, oxygen therapy, or nutritional support); 3) Comparison: patients in the control group received the same routine western treatment as the intervention group; 4) Outcomes: the primary outcomes were clinical symptoms (fever, cough, fatigue, chest tightness, shortness of breath, and expectoration), total efficacy, and adverse events; the secondary outcoms were chest computed tomography manifestations, rate of conversion to severe cases, and fever duration; 5) Study types: randomized controlled trials (RCTs) and case-control studies were included.

Exclusion criteria were as follows: 1) the patient has congenital respiratory disease, asthma, severe interstitial lung disease and other chronic respiratory diseases requiring maintenance therapy; 2) case reports, case series, duplicate reports, letters to editors, comments, and author responses; 3) the full text of the study could not be available.

### 2.3 Data Extraction and Quality Assessment

Two researchers (YPL and ZJZ) independently reviewed the studies and extracted the data. Disagreements were resolved through discussion or consultation with the third researchers (NYL). A standardized data form was used to extract the following information: author name, year of publication, country, period and follow-up time, reference materials, study design, sample size, outcome variables, and so on. Two researchers cross-check the data to ensure accuracy after extraction.

We assessed the quality of included RCTs through the risk of bias assessment tool provided by the Cochrane collaboration (Sterne et al., 2016). The risk grade was judged as low risk of bias, unclear risk of bias, and high risk of bias. The Newcastle-Ottawa Scale (NOS) recommended by the Cochrane Collaboration was used to evaluate the methodological quality of the included case-control studies (Stang, 2010). The evaluation content includes three parts: selection, comparability, and exposure/outcome. Quality assessments were performed by two researchers (YPL and NYL), any discrepancies existed two authors were solved by discussion or consensus.

### 2.4 Statistical Analysis

Statistical analyses were conducted used the RevMan software (version 5.3.5) to calculate the risk ratio (RR), and 95% confidence interval (CI) of dichotomous variables. Standardized mean differences or weighted mean differences with 95% CI were used for the continuous variables. The Mantel-Haenszel method was used for dichotomous variables, while the DerSimonian and Laird inverse variance method were used for continuous variables. Heterogeneity between included studies was assessed by the *I* ^2^ statistic and *p* values. *I* ^2^ ≤ 50% were considered to have little heterogeneity; *I* ^2^ > 50% were considered to have substantial heterogeneity (Higgins et al., 2003). When heterogeneity cannot readily be explained, one analytical approach is to incorporate it into a random-effect model. Otherwise, a fixed-effect model was used. Forest plots were generated to show summarized results. A sensitivity analysis was performed to identify sources of heterogeneity. The Begg’s rank correlation test or Egger’s linear regression test was performed to quantize the publication bias if the number of included studies exceeded nine.

## 3 Results

### 3.1 Literature Search

**FIGURE 1:**
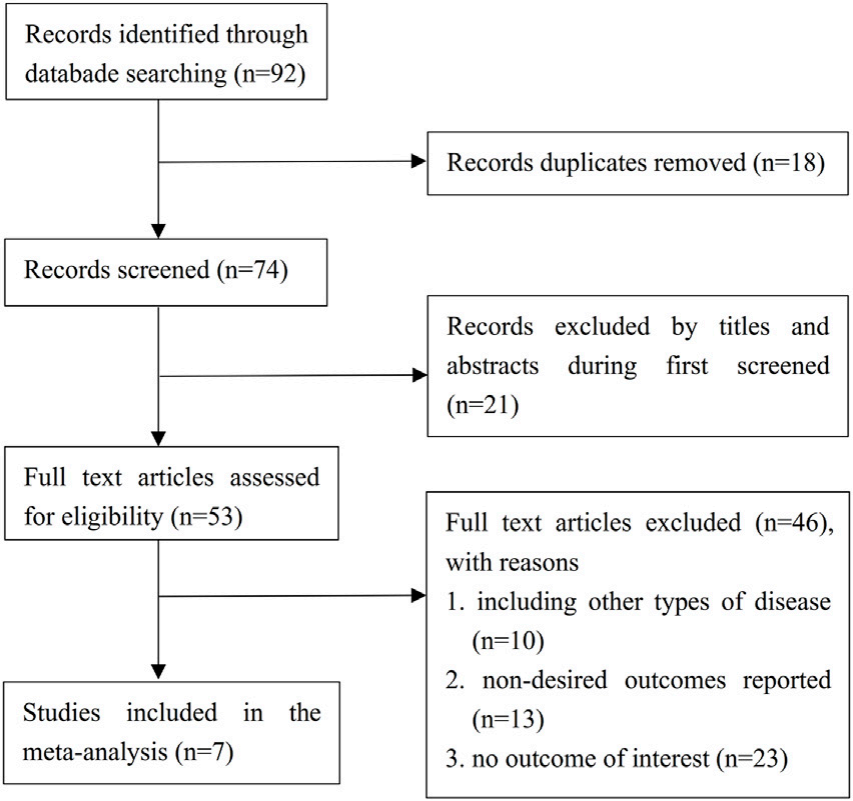
Flow diagram of the literature selection process. RCTs = randomized controlled trials.

A total of 92 records were retrieved from all databases. After the removal of 18 duplicate citations, 74 records were filtered based on the title and abstract. Twenty-one of them excluded for unrelated topics. We reviewed the full text of the remaining 53 studies, identified 7 studies that met the inclusion criteria for meta-analysis (Cheng et al., 2020; Liu et al., 2020; Lv et al., 2020; Wang et al., 2020; Yao et al., 2020; Yu et al., 2020; Hu et al., 2021). The detailed PRISMA flowchart describing the literature search process was demonstrated in Figure 1.

### 3.2 Study Characteristics

Sever studies involving 916 participants contributed to the meta-analysis. All research comes from China. Three studies were RCTs (Wang et al., 2020; Yu et al., 2020; Hu et al., 2021) and the rest were case-control studies (Cheng et al., 2020; Liu et al., 2020; Lv et al., 2020; Yao et al., 2020). One of the included studies came from the English database (Hu et al., 2021) and 6 from the Chinese database (Cheng et al., 2020; Liu et al., 2020; Lv et al., 2020; Wang et al., 2020; Yao et al., 2020; Yu et al., 2020). The sample size ranges from 32 to 295, and the percentage of females ranges from 38.3 to 66.7. The average age of the participants was between 44.06 (SD: 14.23) and 62.4 (SD: 12.3). The diagnostic criteria included in those studies were based on the Protocol for Diagnosis and Treatment of Novel Coronavirus Pneumonia issued by the Chinese government, while the versions were different. In terms of interventions, all treatment groups were used LQ combined with routine western treatment (such as antiviral medications, respiratory assisted ventilation, anti-infection, glucocorticoid therapy, and symptomatic therapies), while all the control group was used the routine western treatment. Except for 2 studies that did not specify the treatment duration (Liu et al., 2020; Yao et al., 2020), the other studies ranged from 7 to 14 days (Table 1).

**TABLE 1:** Description of included studies and patient characteristics.

Study	Location	Study type	Sample size	Gender (%F)	Age [mean (SD)]	Diagnostic criteria	Intervention	Dose (LQ)/Duration (days)	Outcomes	NOS
Yao et al. 2020 ^23^	China	case-control	42	66.7	T: 57.1 ± 14.0 C: 62.4 ± 12.3	RT-PCR assay for throat swab specimens	T: LH combined with conventional treatment C: conventional treatment	1 bag, Tid/NA	③⑥⑦⑧⑨⑩⑪⑫	7
Cheng et al. 2020 ^2^	China	case-control	102	60.0	T: 55.5 ± 12.3 C: 55.8 ± 11.6	RT-PCR assay for throat swab specimens	T: LH combined with conventional treatment C: conventional treatment	6 g, Tid/7	②③④⑤⑥⑦⑧⑨⑩⑪⑫	7
Wang et al. 2020 ^19^	China	RCT	60	38.3	T: 28–69 C: 29–68	RT-PCR assay for throat swab specimens	T: LH combined with conventional treatment C: conventional treatment	4 pieces, Tid/10		NA
Yu et al. 2020 ^24^	China	RCT	295	58.0	T: 48.27 ± 9.56 C: 47.25 ± 8.67	RT-PCR assay for throat swab specimens	T: LH combined with conventional treatment C: conventional treatment	6 g, Tid/7	① ⑤	NA
Hu et al. 2021 ^6^	China	RCT	284	52.8	T: 50.4 ± 15.2 C: 51.8 ± 14.8	RT-PCR assay for throat swab specimens	T: LH combined with conventional treatment C: conventional treatment	4 capsules, Tid/14	①②③④⑤	NA
Liu et al. 2021 ^8^	China	case-control	32	53.1	T: 49.85 ± 17.10 C:44.06 ± 14.23	RT-PCR assay for throat swab specimens	T: LH combined with conventional treatment C: conventional treatment	4 capsules, Tid/NA	②③⑤	5
Lv et al. 2020 ^12^	China	case-control	101	45.5	T: 59.1 ± 16.56 C: 60.2 ± 17.01	RT-PCR assay for throat swab specimens	T: LH combined with conventional treatment C: conventional treatment	6 g, Tid/10	②④⑤⑥⑦⑧⑨⑩⑪⑫	7

RCT: randomized controlled trial; LH: lianhuaqingwen; T: treatment group; C: control group; RT-PCR: reverse transcription-polymerase chain reaction; NA: not available; F: female; ①Total efficacy; ②Adverse events; ③Fever duration; ④Chest CT, manifestations; ⑤Aggravation rate; ⑥fever; ⑦cough; ⑧shortness of breath; ⑨fatigue; ⑩chest tightness; ⑪expectoration; ⑫poor appetite.

### 3.3 Quality of Assessment

**FIGURE 2:**
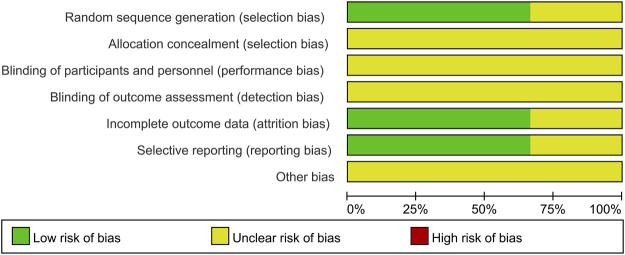
Risk of bias graph presented as percentage across all studies (green represents low risk of bias; red represents high risk of bias, and yellow represents an unclear risk of bias).

For the three RCTs, two studies specified the random sequence generation (Hu et al., 2021; Yu et al., 2020). It is hard to judge the method of allocation concealment and blinding based on limited data. One study cannot judge incomplete outcome data and selective reporting (Wang et al., 2020). Overall, the quality of the included RCTs was judged to be low (Figure 2). Four case-control studies with participants from two cohorts used the NOS scale to assess the quality of the study. The highest score is 7, and the lowest score is 5. All cases in the control group came from the corresponding hospital. Only one study did not report the comparability (Liu et al., 2020). None of the studies reported the no-response rate. Overall, these studies indicated a moderate quality (Table 1).

### 3.4 Meta-Analysis of Primary Outcomes

#### 3.4.1 Clinical Symptoms

The Protocol for Diagnosis and Treatment of Novel Coronavirus Pneumonia which was issued by the Chinese government sets fever, cough, and fatigue as the primary clinical symptoms due to it is common in most COVID-19 pneumonia patients. Three of the 7 studies monitored these symptoms from baseline to endpoint (Cheng et al., 2020; Lv et al., 2020; Yao et al., 2020). Overall, compared with control group, the treatment group was more potential to improve fever (OR = 3.43, 95%CI 1.78, 6.59), cough (OR = 3.39, 95%CI 1.85, 6.23), and fatigue (OR = 2.82, 95%CI 1.44, 5.53). No heterogeneity in all pooled results (*I* ^2^ = 0%) (Table 2). We also conducted a meta-analysis of other clinical symptoms. The pooled results demonstrated that the treatment group was superior to control group in relieving chest tightness (OR = 3.02, 95%CI 1.23, 7.42), shortness of breath (OR = 10.13, 95%CI 3.69, 27.79), and expectoration (OR = 4.30, 95%CI 1.01, 18.22) (Table 2).

**TABLE 2:** Comparison of the disappearance rate of clinical symptoms.

Clinical symptoms	No. of studies	Statistical method	Pooled estimate value		Heterogeneity
			RR (95% CI)	*P*-Vaule	*I* ^*2*^ *P*-Vaule
Fever	3	Fixed	1.49 (1.20, 1.83)	0.0002	0% 0.63
Cough	3	Fixed	1.67 (1.26, 2.21)	0.0003	0% 0.84
Fatigue	3	Random	1.65 (0.75, 3.65)	0.21	53% 0.12
Chest tightness	3	Random	2.00 (0.81, 4.96)	0.13	64% 0.06
Shortness of breath	3	Fixed	3.82 (1.92, 7.61)	0.0001	0% 0.79
Poor appetite	3	Random	2.52 (0.46, 13.83)	0.29	89% 0.0002
Expectoration	3	Random	2.46 (0.81, 7.51)	0.11	69% 0.04

### 3.5 Total Efficacy

**FIGURE 3:**
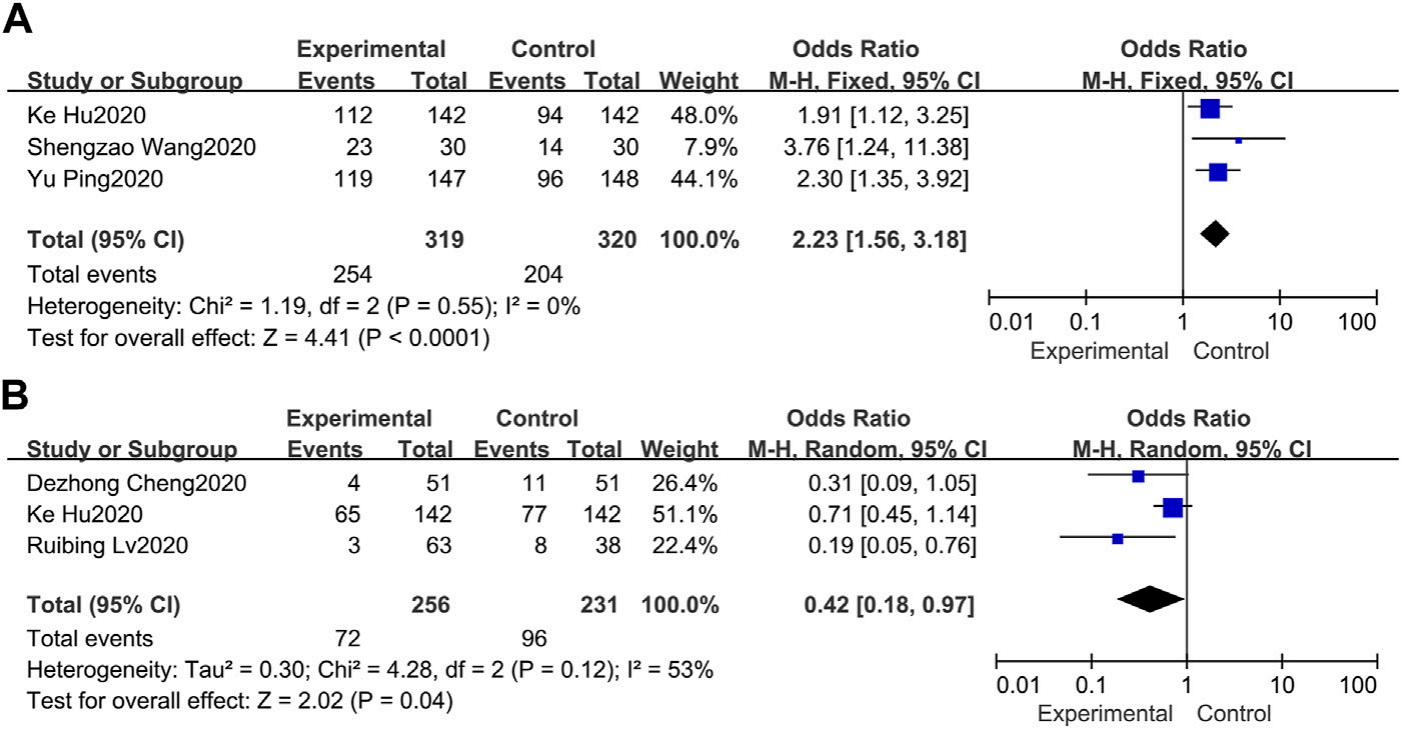
Forest plots of the pooled results of the meta-analysis for total efficacy **(A)** and adverse events **(B)**.

Total efficacy is defined as the ratio of significant effect cases and partially effective cases to the total number of cases. Three of the 7 studies set total efficacy as the primary endpoint (Wang et al., 2020; Yu et al., 2020; Hu et al., 2021). In terms of the three studies, the minimum percentage of the total efficacy was 76.67, and the maximum percentage was 91.50 in the treatment group. Overall, our meta-analysis in a fixed-model showed that the total efficacy in the treatment group was superior to the control group (OR = 2.23, 95%CI 1.56, 3.18) with no heterogeneity (*I* ^2^ = 0%) (Figure 3A).

### 3.6 Adverse Events

Three of the 7 studies recorded adverse events (Cheng et al., 2020; Hu et al., 2021; Lv et al., 2020). The pooled results of the 3 studies indicated that routine western treatment paired with LQ could significantly decrease the occurrence of adverse events (OR = 0.42, 95%CI 0.18, 0.97) (Figure 3B). For heterogeneity (*I* ^2^ = 53%), we conducted a sensitivity analysis. Through excluded the study of hu et al. (Hu et al., 2021) due to its prospective with a comprehensive record of adverse events, leading to heterogeneity reduced to 0%, while the overall effect estimate was not altered substantially.

### 3.7 Meta-Analysis of Secondary Outcomes

#### 3.7.1 Fever Duration

**FIGURE 4:**
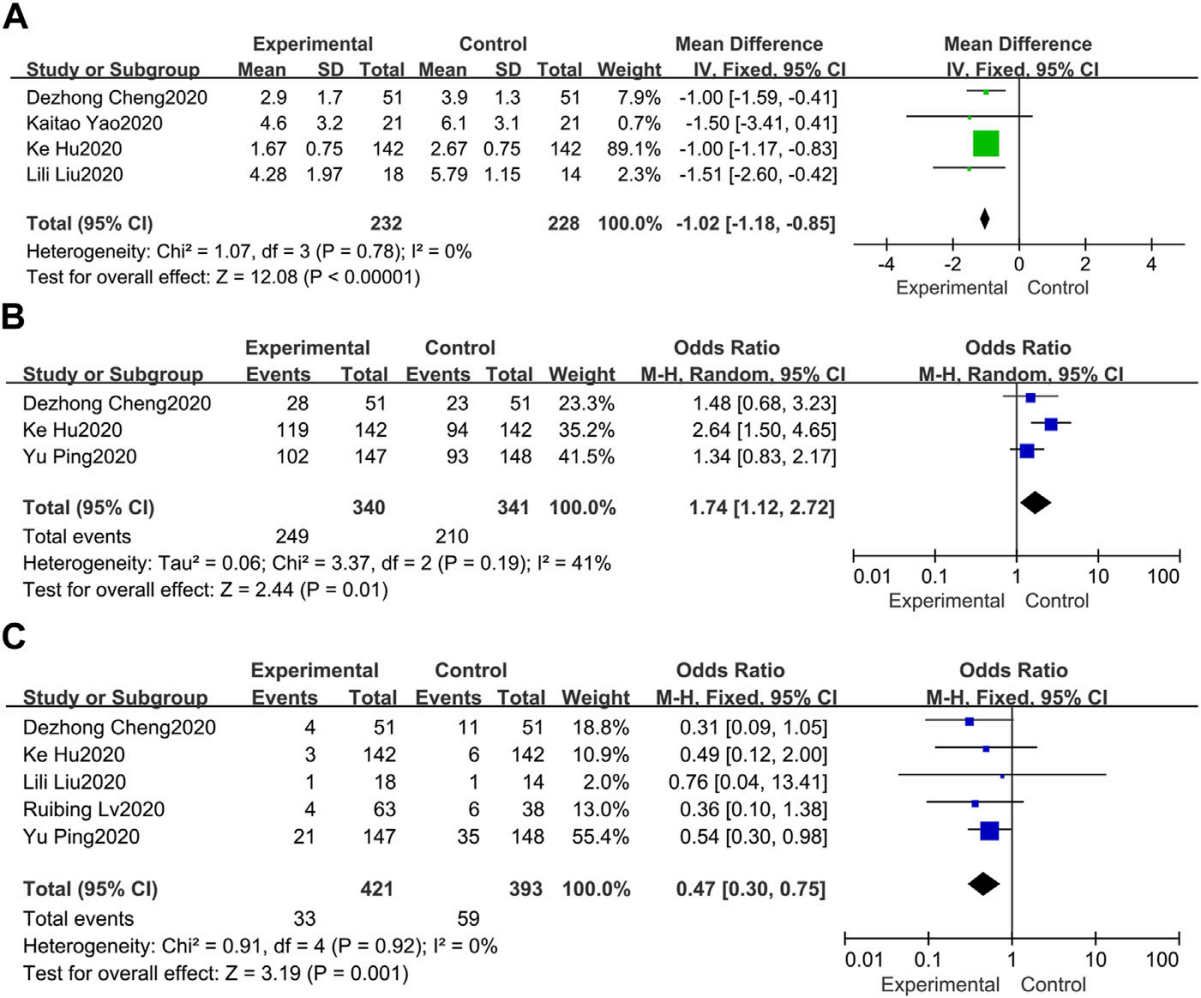
Forest plots of the pooled results of the meta-analysis for the fever duration **(A)**, chest CT manifestations **(B)**, and rate of conversion to severe cases **(C)**.

As far as the duration of fever is concerned (Cheng et al., 2020; Liu et al., 2020; Yao et al., 2020; Hu et al., 2021), the pooled results of 4 available data in a fixed-effect model suggested that treatment group can significantly shorten the duration of fever for inpatients compared with the control group (MD = −1.02, 95%CI −1.18, −0.85) (Figure 4A). In the meantime, no heterogeneity between these studies was revealed (*I* ^*2*^ = 0%).

### 3.8 Chest CT Manifestations

Chest CT improvement is generally defined as the visible absorption of pulmonary infiltrates. Three of 7 studies documented changes in chest CT (Cheng et al., 2020; Yu et al., 2020; Hu et al., 2021), indicating that the absorption of pulmonary infiltrates in the treatment group was more obvious than the control group (OR = 1.74, 95%CI 1.12, 2.72) (Figure 4B). Besides, mild heterogeneity was observed (*I* ^*2*^ = 41%).

### 3.9 Rate of Conversion to Severe Cases

Five of 7 studies evaluated the rate of conversion to severe cases (Cheng et al., 2020; Liu et al., 2020; Lv et al., 2020; Yu et al., 2020; Hu et al., 2021). Overall, the pooled OR in a fixed-effect model demonstrated that the rate of conversion to severe cases in the treatment group was lower than the control group at the endpoint (OR = 0.47, 95%CI 0.30, 0.75) (Figure 4C). Besides, no heterogeneity was observed in these included studies (*I* ^*2*^ = 0%).

### 3.10 Publication Bias

Publication bias did not evaluate due to the number of studies in any comparative analysis was less than 10.

## 4 Discussion

This comprehensive meta-analysis was based on 7 trials, including 916 participants assigned to available LQ combined with routine western treatment or routine western treatment alone to compare efficacy and safety. The sample size, primary endpoint, and duration vary between different studies. The results suggested that for COVID-19 patients, LQ combined with western medicine treatment was more efficacious with clinical symptoms improvements than western medicine treatment. In addition, LQ combined with western medicine has advantages in shortening fever time, decreasing the incidence of adverse reactions, and reducing the rate of severe case conversion. In the current lack of effective treatment therapies, our study shown LQ combined with routine western medicine treatment is an effective strategy for relieving the clinical symptoms of the COVID-19 in consideration of safety.

Since the outbreak of COVID-19, research institutions and companies from various countries have accelerated the development of effective medicine, however, no drug or vaccine approved. Several candidates may be envisaged to control or prevent COVID-2019 infections, including vaccines, monoclonal antibodies, oligonucleotide-based therapies, peptides, interferon therapy, and small molecule drugs (Lu, 2020). However, new interventions may be difficult in the short term. Given the urgency of the outbreak, the WHO has initiated a clinical trial in Norway and Spain to evaluate four of the most promising anti-COVID-19 drugs, including chloroquine, hydroxychloroquine, remdesvir, and Lopi Navir/ritonavir (lopinavir/ritonavir, Kreiz) (Gu et al., 2020; Lan et al., 2020). These drugs were tested individually or in combination, and we believe the results will be as expected. Besides, two other infectious diseases caused by a human coronavirus, SARS, and the Middle East respiratory syndrome also provide a reference (Gu et al., 2020).

LQ has been marketed for more than 10 years since the outbreak of SARS in 2003 in China. The main ingredients of LQ include Forsythia suspensa (Thunb.) Vahl [Oleaceae; Dried fruit], Lonicera japonica Thunb. [Caprifoliaceae; Dried flower bud or opening flower], Ephedra sinica Stapf [Ephedraceae; Dried herbaceous stem], Isatis tinctoria subsp. tinctoria [Brassicaceae; Dried root], Pogostemon cablin (Blanco) Benth. [Lamiaceae; Dried aerial part], Rheum palmatum L. [Polygonaceae; Dried root and rhizome], Rhodiola crenulata (Hook.f. & Thomson) H.Ohba [Crassulaceae; Dried root and rhizome], Dryopteris crassirhizoma Nakai [Polypodiaceae; Dried rhizome and frond bases], Houttuynia cordata Thunb. [Saururaceae; Dried aerial part], Prunus armeniaca L. [Rosaceae; Dried ripe seed], Glycyrrhiza uralensis Fisch. ex DC. [Fabaceae; Dried root and rhizome], Menthol [C_10_H_20_O] and Gypsum [CaSO_4_·2H_2_O] (Hu et al., 2021). The amount of each drug in a polyherbal preparation is presented in Supplementary Table S1. Previous studies have shown that LQ could inhibit the release of tumor necrosis factor-α, interleukin-6, macrophage chemokine protein-1, and inducible protein 10 to alleviate the lung injury associated with inflammatory cell infiltration. Further, clinical studies have confirmed that LQ also has a therapeutic effect on influenza A virus infection (Zhao et al., 2014). These findings provide evidence for the clinical application of LQ in COVID-19. The latest data supports this view; LQ significantly inhibited SARS-CoV-2 replication in Vero E6 cells and markedly reduced pro-inflammatory cytokines production at the mRNA levels, indicating that patients with COVID-19 could recommend LQ to reduce the burden of clinical symptoms (Runfeng et al., 2020).

Several exciting pieces of evidence were found in our research, but as a new type of respiratory disease, COVID-19 has many unknown factors yet to be resolved. Besides, the duration of our included studies is shorter (up to 14 days), however, COVID-19 may require a longer follow-up time to better assess the efficacy and possible adverse reactions. Although we emphasize the advantages of LQ in the treatment of COVID-19, some routine treatments of Western medicine such as antiviral medications, respiratory assisted ventilation, anti-infection, and symptomatic therapies are still indispensable. The purpose of this research is to provide a more effective and safe treatment method to rescue patients from disease as soon as possible.

Considering the novelty of COVID-19, we urgently need large-scale, multi-dimensional findings to understand the efficacy and safety of LQ against SARS-CoV-2. Consistent with the results of several recently published related meta-analyses, the present study showed that LQ combined with western medicine was superior to western medicine alone in improving clinical symptoms. Liu et al. (2020) included 3 case-control studies. On this basis, we included the study of Lv et al. (2020) with a NOS score of 7 (sample size = 101). At the same time, our report also provides the aggravation rate of conversion to severe cases and the results of the intervention group in improving clinical symptoms (fever, cough, fatigue, chest tightness, shortness of breath, expectoration) and shortening the duration of fever. In the meta-analysis of Wang et al. (2022), the result of Wang et al. (2020) was not included in the overall effective rate, and the study did not report chest computed tomography manifestations and the aggravation rate of conversion to severe cases. In Zeng et al. (2020) ‘s study, only 2 studies were included. Although similar meta-analyses exist, we hope that this study, together with those results, can provide multi-dimensional information on the efficacy and safety of LQ in COVID-19.

Limitations in current systematic review and meta-analysis were inevitable. First, we carefully evaluated the quality of the included RCTs found that most were considered high-risk in terms of selection bias, especially allocation concealment. High risks and uncertain risks were identified in the blinding of participants, personnel, and outcome assessors. However, considering the urgency of the outbreak, these studies were not rigorous enough in design. Second, although we comprehensively and systematically included eligible studies, the sample size of some studies was still small. Third, not all studies involve data with pre-determined outcomes, however, our meta-analysis includes all eligible studies to reduce the possibility of bias. Fourth, publication bias was not monitored due to the fewer studies. Fifth, LQ dosage form and treatment time are inconsistent, and there are differences in actual intake. Finally, all the original study participants had a large age span and were from China (with significant individual differences in age and ethnicity), which may have an impact on the outcomes. Nonetheless, given the uniform distribution of patient characteristics in each treatment group and the objective methods used, our analysis still provides an objective assessment of uncertainties.

## 5 Conclusion

In conclusion, our findings indicate that LQ combined with routine western medicine treatment may be an effective strategy for relieving the clinical symptoms of theCOVID-19 in consideration of safety. However, due to the urgency of SARS-CoV-2 outbreaks leading to low methodological quality and not rigorous designs. This meta-analysis cannot draw clear conclusions. Therefore, more standardized RCTs are needed to verify the above conclusion. In the future, such research should be conducted following the requirements of evidence-based medicine.

## Data Availability

The raw data supporting the conclusion of this article will be made available by the authors, without undue reservation.
