# Supplementary material for: Efficacy and Safety of Chinese Medicine Lianhua Qingwen for Treating COVID-19: An Updated meta-Analysis
Source: Front Pharmacol. 2022 Jun 3;13:888820. doi: 10.3389/fphar.2022.888820 (PMC9204491; doi:10.3389/fphar.2022.888820)
Supplement: Supplementary file 1 [file Table1.docx]

| **Table 1.** Formulation of Lianhua Qingwen capsule (Granule) | | | |
| --- | --- | --- | --- |
| **Drug name** | **Species** | **Components** | **%** |
| Forsythia suspensa (Thunb.) Vahl | Oleaceae | Dried fruit | 12.70 |
| Lonicera japonica Thunb. | Caprifoliaceae | Dried flower bud or opening flower | 12.70 |
| Ephedra sinica Stapf | Ephedraceae | Dried herbaceous stem | 4.20 |
| Isatis tinctoria subsp. Tinctoria | Brassicaceae | Dried root | 12.70 |
| Pogostemon cablin (Blanco) Benth. | Lamiaceae | Dried aerial part | 4.20 |
| Rheum palmatum L. | Polygonaceae | Dried root and rhizome | 2.50 |
| Rhodiola crenulata (Hook.f. & Thomson) H.Ohba | Crassulaceae | Dried root and rhizome | 4.20 |
| Dryopteris crassirhizoma Nakai | Polypodiaceae | Dried rhizome and frond bases | 12.70 |
| Houttuynia cordata Thunb. | Saururaceae | Dried aerial part | 12.70 |
| Prunus armeniaca L. | Rosaceae | Dried ripe seed | 4.20 |
| Glycyrrhiza uralensis Fisch. ex DC. | Fabaceae | Dried root and rhizome | 4.20 |
| Menthol |  | C_10_H_20_O | 0.40 |
| Gypsum |  | CaSO_4_·2H_2_O | 12.70 |
